# Can project-based learning improve students’ mathematics learning outcomes: a three-level meta-analysis

**DOI:** 10.3389/fpsyg.2026.1868415

**Published:** 2026-09-04

**Authors:** Chunting Wang, Wenlan Zhang, Jiaxin Cheng

**Affiliations:** 1 School of Teacher Development, Shaanxi Normal University, Xi’an, Shaanxi, China; 2 Xi'an Academy of Educational Science, Xi’an, China; 3 Faculty of Education, Shaanxi Normal University, Xi’an, China

**Keywords:** cognitive abilities, mathematical learning outcomes, non-cognitive abilities, project-based learning, three-level meta-analysis

## Abstract

Against the backdrop of the worldwide shift toward competency-driven basic education reform, project-based learning (PBL) is widely recognized for its student-centered design and authentic problem-solving approach. Empirical evidence on the effectiveness of Mathematics PBL is highly varied; traditional meta-analysis often fails to properly address nested data structures. This study conducted a three-level meta-analysis spanning the past 20 years, a total of 35 eligible studies were included. In the primary meta-analytic model, two statistically outlying studies were identified and excluded based on Cook’s distance. Ultimately, 33 studies with 84 effect sizes were incorporated into the main effect analysis.to comprehensively quantify PBL’s impact on students’ mathematical achievement. The results indicated a significant overall effect size for PBL (Cohen’s *d* = 0.529, 95% CI [0.38, 0.68]), but publication bias analyses suggested potential overestimation (trim-and-fill corrected estimate = 0.275); therefore, findings should be interpreted cautiously. Moderator analyses revealed that cultural background and intervention duration significantly influenced outcomes. Notably, PBL demonstrated strong effectiveness within a 1-to-8-week timeframe. Non-cognitive abilities showed a numerically larger effect than cognitive abilities, but the difference was not statistically significant (*p* = 0.083). No significant moderators were found for educational level, class size, group size, mathematics knowledge domain, publication type, or the number of technology types used, suggesting that PBL is broadly generalizable across various mathematics teaching contexts. Given the presence of publication bias, these results provide moderate rather than robust support for the localized application of mathematics PBL.

**Systematic review registration:**

https://www.crd.york.ac.uk/PROSPERO/view/CRD420251105265, identifier CRD420251105265.

## 1 Introduction

Mathematics is a logically rigorous and application-oriented discipline that emphasizes abstract reasoning, logical deduction, active knowledge construction and authentic problem-solving (Lang-Raad, 2025). Unlike subjects that rely heavily on rote memorization, mathematics learning focuses on contextual comprehension and inquiry-based practice, which places high demands on learners’ cognitive processing, behavioral engagement and interpersonal interaction (Rijken and Fraser, 2023; Lazić et al., 2021). Against the backdrop of competency-oriented reforms in global basic education (Zhang and Hu, 2019), lecture-based instruction and repetitive drills may lead to fragmented and decontextualized knowledge acquisition, as well as passive student engagement in class (Rehman et al., 2023). Social cognitive theory argues that learning effectiveness arises from the dynamic interaction of three elements: individual cognition, behavioral engagement and external environment (Bandura, 1991). Devoid of concrete contextual tasks and regular peer collaboration, traditional mathematics classrooms may undermine students’ academic self-efficacy and discourage their proactive learning engagement (Rijken and Fraser, 2023). In addition, achievement goal theory suggests that the quality of learning is strongly associated with learners’ goal orientations (Anderman and Patrick, 2012). When instruction places excessive emphasis on repetitive drills and exam-oriented assignments, students may tend to develop a performance goal orientation, which could possibly reduce their intrinsic motivation and hinder deep learning in mathematics (Rijken and Fraser, 2023).

Against this backdrop, project-based learning (PBL) emerges as a promising student-centered approach to address the above instructional challenges (Lazić et al., 2021; Wijnia et al., 2024). By embedding mathematical knowledge in authentic thematic problem-solving tasks (Lazić et al., 2021; Saparbayeva et al., 2025), PBL promotes students’ active knowledge construction, collaborative inquiry, and reflective practice (Lin et al., 2025). This learning model integrates individual cognition, learning behaviors, and social interaction, and prioritizes in-depth knowledge exploration over mechanical rote training (Rijken and Fraser, 2023). Given these theoretical and pedagogical strengths, both the U.S. Common Core State Standards and China’s 2022 Compulsory Education Mathematics Curriculum Standards advocate PBL implementation to advance competency-based mathematics education.

Nevertheless, empirical evidence regarding PBL effectiveness in mathematics education remains inconsistent. Compared with other disciplines, empirical research on mathematical PBL is relatively scarce and presents substantial cross-study heterogeneity. Multiple studies have verified that PBL can improve students’ mathematical achievement, core competencies, and learning attitudes (Alenezi, 2023; Lazić et al., 2021; Rehman et al., 2023), whereas other studies report insignificant or even negative PBL effects in specific instructional scenarios (Rijken and Fraser, 2023; Tabuk and Özdemir, 2009). Such conflicting findings mainly result from differences in research samples, intervention designs, implementation contexts, and measurement tools, leaving the generalizable effects of mathematical PBL insufficiently clarified. These inconsistencies arise from variations in research participants, intervention duration, project design, implementation contexts, methodological approaches, sample sizes, and assessment measures, making it challenging to accurately determine the true effectiveness of mathematics PBL.

Methodologically, conventional two-level meta-analysis cannot adapt to the nested data structure prevalent in PBL empirical studies, as multiple effect sizes can be extracted from a single primary study. Ignoring within-study data dependence may cause statistical bias and unstable pooled results. In contrast,three-level meta-analysis is a statistical method designed to handle nested data structures. It facilitates a comprehensive synthesis of multiple research findings by accounting for within-study, between-study, and higher-level variations, thereby producing more precise estimates of overall effect sizes (Nie et al., 2025; Van den Noortgate et al., 2012). Accordingly, this study employs a three-level meta-analytic approach to systematically synthesize both domestic and international empirical literature. Its aims are to clarify the overall effect of PBL on students’ mathematics achievement, identify key moderating factors, address methodological limitations of existing meta-analyses, and provide more rigorous empirical evidence to inform mathematics teaching practices and educational reform.

## 2 Literature review

### 2.1 Definition of project-based learning

PBL is defined as an instructional approach featuring: (a) a driving question that organizes and drives project activities; (b) sustained inquiry over an extended period; (c) student autonomy in planning, executing, and reflecting; (d) collaboration among students; and (e) a public product or artifact. These features distinguish PBL from case-based learning (typically case-oriented without a multi-stage project), problem-based learning (short-term, problem-focused), and realistic mathematics education (situated applications but no complete project cycle) (Alenezi, 2023; Lin et al., 2025). The development of PBL is grounded in robust theoretical foundations: Kolb’s experiential learning theory provides methodological guidance for the inquiry process in PBL, while constructivism (Piaget and Vygotsky) establishes the core logic of student agency and active knowledge construction (Sancar-Tokmak and Dogusoy, 2023). By creating authentic problem contexts, PBL encourages learners to engage actively in inquiry, develop problem-solving and critical-thinking skills through collaborative interaction, and move beyond the limitations of passive reception characteristic of traditional instruction (Novalia et al., 2025).

### 2.2 Application of project-based learning in mathematics education

In mathematics education, PBL effectively bridges abstract mathematical concepts and real-world applications, enabling learners to deepen their understanding and apply mathematical knowledge while solving authentic problems, thereby avoiding rote memorization and formalistic learning (Rijken and Fraser, 2023). This approach aligns closely with the competency-oriented core requirements of modern mathematics education. In recent years, a substantial body of empirical research has examined the impact of PBL on students’ mathematics learning. Synthesizing existing studies, the effects of PBL on mathematics learning can be systematically categorized into cognitive and non-cognitive dimensions.

At the cognitive level, research confirms that PBL significantly enhances students’ problem-solving skills, knowledge transfer, and mathematical application abilities (Saparbayeva et al., 2025; Rehman et al., 2023; Alenezi, 2023). By encouraging students to engage proactively in problem-solving (Faulkner et al., 2023), PBL deepens their understanding of mathematical concepts and theorems, helps them construct systematic knowledge networks (Boaler, 2002), links abstract concepts to real-world applications, and fosters higher-order thinking and mathematical competence (Saparbayeva et al., 2025). However, some studies report non-significant effects of PBL on mathematics achievement (Rijken and Fraser, 2023; Tabuk and Özdemir, 2009).

At the non-cognitive level, studies indicate that PBL significantly enhances learning interest, motivation, classroom engagement, and self-efficacy, while effectively reducing mathematics anxiety (Alenezi, 2023; Holmes and Hwang, 2016). However, some research reports non-significant effects on non-cognitive outcomes (Zhang and Ma, 2023; Chen and Yang, 2019). Overall, the effectiveness of PBL may be moderated by factors such as participant characteristics, content, intervention duration, and implementation quality.

### 2.3 Previous meta-analyses

Several meta-analyses and systematic reviews have synthesized the effects of PBL in education, demonstrating positive impacts on academic achievement (Chen and Yang, 2019; Zhang and Hu, 2019; Zhang and Ma, 2023). However, few studies focus specifically on mathematics. Some research indicates that computer-assisted technology in PBL environments significantly improves mathematics achievement among Indonesian students (Juandi et al., 2025). Nevertheless, most prior meta-analyses employ traditional two-level models, which only account for the hierarchical structure of effect sizes nested within studies and overlook the common occurrence of multiple correlated effect sizes (e.g., measuring different outcome variables) within single studies in PBL research. This limitation can bias parameter estimation—such as producing inaccurate standard errors—fail to distinguish within- and between-study variance, and reduce the accuracy of moderator identification and the reliability of conclusions. The three-level meta-analytic model addresses these issues by decomposing variance in effect sizes into sampling error, within-study variance (Level 2), and between-study variance (Level 3), thereby improving estimation accuracy and fully leveraging the data’s value.

### 2.4 Moderating variables

Cognitive abilities refer to forms of cognition and awareness that cover academic achievement and problem‑solving skills. Non‑cognitive abilities consist of learning motivation, attitudes, and interests (Yang et al., 2025). Existing research indicates that PBL significantly enhances learning outcomes, particularly academic achievement (Zhang and Ma, 2023). Mathematics learning outcomes encompass not only grades but also attitudes and motivation. Analyzing cognitive and non-cognitive outcomes separately offers a more comprehensive understanding of PBL’s impact on mathematics learning.

Cultural background strongly influences mathematics learning. Students in societies with a strong long-term orientation demonstrate significantly better mathematics achievement than those in short-term oriented societies. Although the individualism–collectivism dimension is non-significant in some studies, it remains an important factor (Hu et al., 2018). Dede et al. (2020) examine the value orientations of mathematical modeling tasks from cross-cultural perspectives, providing empirical evidence linking individualism and collectivism to mathematics education. Chen and Yang (2019) find that school location significantly affects learning outcomes, with stronger effects observed in Europe, North America, and West Asia compared to East Asia. Conversely, Zhang and Ma (2023) report stronger effects of PBL in Asia, especially Southeast Asia, than in Western Europe and North America. Most prior meta-analyses focus on direct geographic effects rather than the underlying cultural mechanisms that shape PBL effectiveness.

Recommendations for the duration of PBL interventions vary across studies. Chen and Yang (2019) found that implementing PBL for more than 2 h per week yields stronger effects than less than 2 h. Zhang and Ma (2023) demonstrated that interventions lasting 9–18 weeks produce the most significant effects, with diminishing returns beyond 18 weeks. Zhang and Hu (2019) reported the weakest effects for durations of 0 to 3 months and the strongest effects for interventions lasting over 6 months, suggesting that longer durations improve achievement. These studies primarily focus on general learning outcomes rather than mathematics-specific effects, highlighting the need for further investigation into the optimal duration of PBL interventions in mathematics.

Educational stage, group size, and class size are important contextual factors. Zhang and Hu (2019) found stronger PBL effects in university and elementary settings than in secondary schools. Zhang and Ma (2023) confirmed advantages for small classes, senior high schools, and groups of 4–5 students. However, other studies have shown non-significant effects of group size, with PBL effects remaining consistent across educational stages and group sizes (Chen and Yang, 2019).

Different types of mathematical knowledge exhibit distinct characteristics: algebra emphasizes logical deduction and symbolic computation; geometry focuses on spatial reasoning and graphical analysis; statistics centers on data interpretation and inductive reasoning; and analytical mathematics is highly abstract, involving rigorous logical sequences. Cognitive demands and learning paradigms vary significantly across these subject areas. Yunita et al. (2022) found that mathematical content moderates the effects of PBL on students’ mathematical problem-solving abilities. Juandi et al. (2025) confirm that content type influences mathematics achievement in PBL environments, with measurement content demonstrating the strongest effects.

Jonassen classifies educational technology into six categories: cognitive tools, productivity tools, information access tools, context-creation tools, communication-collaboration tools, and assessment tools (Zhang and Hu, 2019). Similarly, Zhang and Hu (2019) categorize technology into information access, communication-collaboration, productivity, cognitive/contextual, and assessment/other tools, concluding that a greater variety of technology types enhances the impact of PBL on academic achievement. Additional studies also support the effectiveness of technology-supported PBL (Chen and Yang, 2019). Furthermore, Younas et al. (2025b) conducted a comprehensive investigation of peer-reviewed articles published between 2020 and 2023 and found that the adoption of AI technologies facilitates personalized learning and enhances student engagement. Artificial intelligence tools exert a growing influential effect on students’ learning outcomes (Younas et al., 2025d).

In summary, this study employs a three-level meta-analysis to systematically synthesize empirical literature on PBL in mathematics education, aiming to: (1) estimate the overall effect size of PBL on students’ mathematics learning; (2) compare PBL’s effects on cognitive versus non-cognitive learning outcomes; and (3) identify key moderators of effect sizes to provide rigorous, interpretable evidence for mathematics instruction.

This study addresses two research questions: Question 1: What is the overall effect of PBL on students’ mathematics learning outcomes? Are there differential effects on cognitive versus non-cognitive abilities? Question 2: Do cultural background, intervention duration, educational stage, class size, group size, type of mathematical knowledge, and number of technology types moderate the effect of PBL on students’ mathematics learning outcomes?

## 3 Methods

To ensure systematicity and replicability, this study adhered to the PRISMA 2020 guidelines (Page et al., 2021). The study was prospectively registered in PROSPERO (Registration No.: CRD420251105265).

### 3.1 Literature search

Complete Boolean search strings for each database are provided in Appendix S1. The search was conducted on July 15, 2025 (initial) and updated between December 1, 2025 and January 10, 2026. Literature was searched in both Chinese and English databases. The Chinese databases included CNKI, Wanfang, and CQVIP, using the keywords: (“project-based learning” OR “PBL” OR “PjBL”) AND (“mathematics” OR “math”). English databases included Web of Science, EBSCO, ProQuest, and SpringerLink, supplemented by Google Scholar. The English search string was: (“project-based learning” OR “PBL” OR “PjBL”) AND (“mathematics” OR “math education” OR “mathematics education”). Reference lists of the included studies were hand-searched. The initial search yielded 4,715 records (Web of Science: 839, EBSCO: 1072, ProQuest: 1161, SpringerLink: 1000, CNKI: 614, through references or other ways: 29.)

### 3.2 Study selection

Records were imported into EndNote X21 and screened according to the following criteria: (1) Empirical studies focusing on the effects on mathematics learning outcomes with complete data; theoretical reviews, case studies, and qualitative research were excluded; (2) Studies on project-based learning (PBL) as defined above were included. Studies using problem-based learning (short-term, case-based, without a multi-stage project and public product) were excluded. Studies using integrated STEM frameworks were excluded unless the mathematics effect could be isolated (i.e., mathematics outcomes reported separately from other STEM domains); (3) Participants were from elementary, junior high, senior high, or tertiary education levels; special-needs samples were excluded; (4) Studies reported means (*M*), standard deviations (SD), standard errors (SE), sample sizes, or data convertible to Cohen’s *d* (e.g., *t*, *F*); studies lacking these data were excluded after unsuccessful attempts to contact authors; (5) Duplicate or overlapping publications were excluded, with only the most complete version retained.

**Figure 1:**
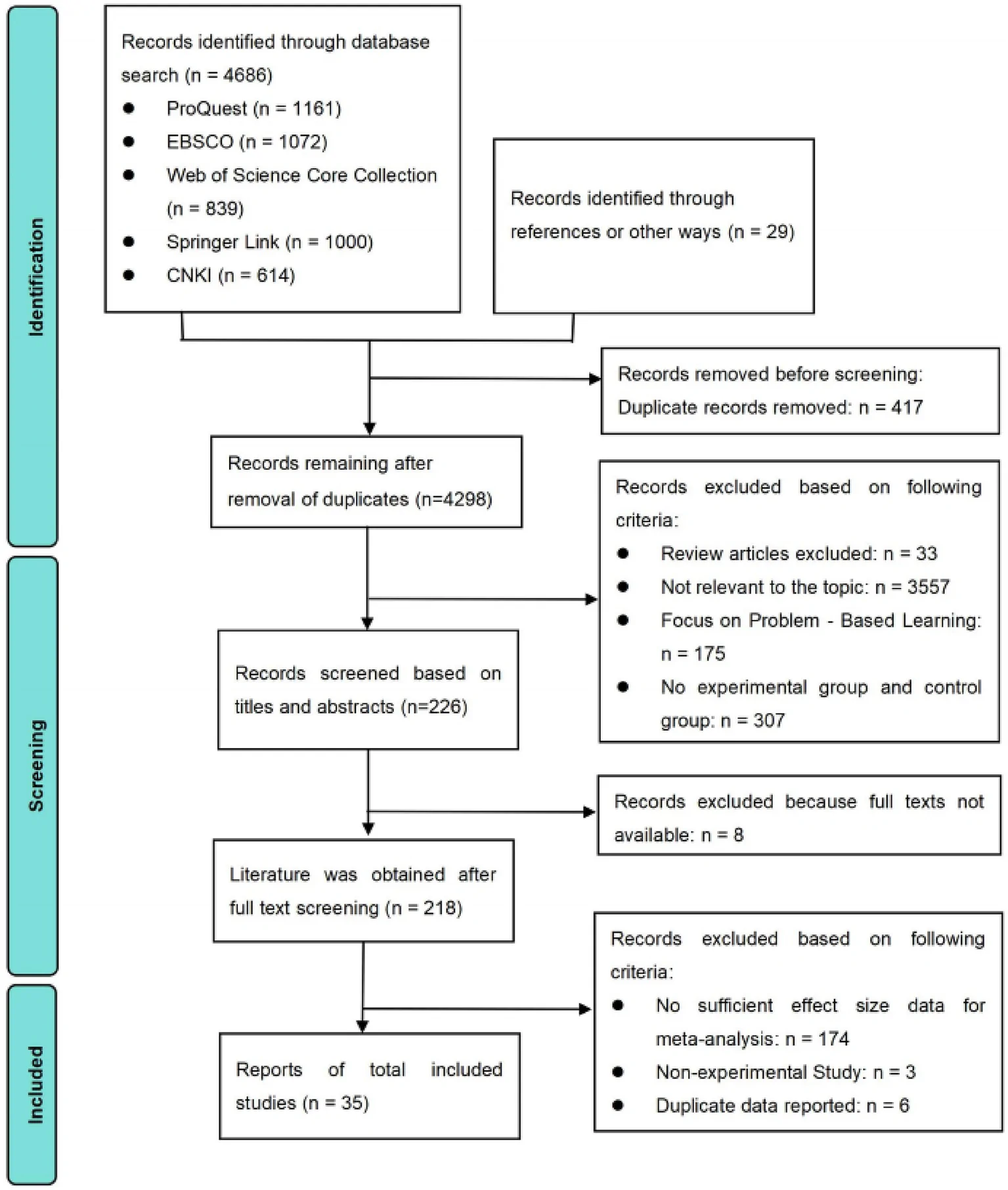
Literature screening workflow diagram (based on PRISMA 2020).

After duplicate removal (*n* = 417), 4,298 records remained. Title/abstract screening excluded 4,072 records, leaving 226 full-text articles. Eight publications were eliminated as their full texts could not be retrieved, resulting in a final dataset of 218 articles. Full-text screening excluded 183 records (reasons: no sufficient effect size data for meta-analysis = 174, non-experimental study = 3, duplicate data reported = 6). Ultimately, 35 studies were included. The PRISMA flow diagram (Figure 1) has been corrected to reflect these exact numbers.

### 3.3 Coding procedure

Two researchers independently coded all studies and reached 100% agreement through discussion. Separate coding was applied to multiple independent samples (e.g., different grades) or multiple effect sizes (e.g., achievement and interest) within a single paper. The coding framework included: (1) basic study information—authors, year, sample size, country/region; (2) moderator variables—cultural background, educational stage, intervention duration, class size, group size, type of mathematical knowledge, and number of technology types. Outcome variables were categorized as cognitive (achievement, problem-solving) and non-cognitive (attitudes, interest, motivation) (see Table 1). A detailed table of included studies (Table 2) is provided, containing country, education level, sample size, intervention duration, outcome type, and number of effect sizes per study. Cultural background was operationalized using Hofstede et al. (2010) Insights individualism–collectivism index (0–100, higher = more individualistic). Each study was assigned the index value of the country where the sample was collected. For the one multinational study, a sample-size-weighted mean was calculated.

**Table 1:** Coding framework.

Dimension	Category	Description	References
Cultural background	Continuous variable	This index ranges from 0 to 100. A higher score indicates a stronger individualistic tendency in the culture, while a lower score represents a stronger collectivistic tendency.	Hu et al. (2018)
Publication type	Journal article	Publication type was coded as a categorical moderator variable, distinguishing between (a) peer-reviewed journal articles and (b) dissertations, theses, and other gray literature, e.g., ((conference proceedings, institutional reports). This classification was used to examine whether the source of publication influenced the pooled effect sizes and to assess the robustness of findings by comparing results across publication types.	
	Dissertation and other materials		
Education level	Primary school	Elementary education (Grades 1–6, ages 6–12)	Chen and Yang (2019)
	Secondary school	Middle and high school education (Grades 7–12, ages 12–18)	
	Tertiary	Post-secondary education (college, university, and graduate levels)	
Intervention duration	Short	1–8 Weeks	Zhang and Ma (2023)
	Medium	9–18 Weeks	
	Long	> 18 Weeks	
Class size	Small	1–30 students	Putri et al. (2025)
	Middle	31–50 students	
	large	>50 students	
Group size	Small	≤5 students	Chen and Yang (2019)
	Large	>5 students	
Type of mathematical knowledge	Algebraic knowledge	The fundamental concepts, principles, and operational rules in the field of algebra.	Juandi et al. (2025)
	Geometric knowledge	Theoretical, conceptual, or applied knowledge related to geometry.	
	Knowledge of probability and statistics	Understanding data handling, graphical representation, descriptive measures, and probabilistic reasoning about chance and uncertainty.	
	Analytical knowledge	Limits, continuity, differentiation, integration, infinite series, and the rigorous foundations of calculus.	
	Others	Content involving the cross-integration of two or more mathematical fields, or comprehensive mathematical knowledge that cannot be clearly attributed to a single branch.	
Technology types	Category 1	Refers to information acquisition tools (Internet, mobile devices, computers, etc.)	Zhang and Hu (2019)
	Category 2	Refers to communication and collaboration tools (QQ, WeChat, Microblog, Blog, etc.)	
	Category 3	Refers to efficiency-enhancing tools (PPT, Flash, electronic whiteboard, online platform, etc.)	
	Category 4	Mind mapping tools, virtual environments, etc.	
	Category 5	Robots, voting systems, discipline-specific instruments and equipment, etc.	
Learning performance	Cognitive abilities	Knowledge mastery, academic performance, problem-solving ability, expression and communication ability	Yang et al. (2025)
	Non-cognitive abilities	Digital literacy, learning motivation, learning engagement, learning interest, self-efficacy	

**Table 2:** A detailed table of included studies.

Author (year)	PT	*N*	*N*	CB	Country	EL	InD	CS	GS	TMK	NTT	TLP	
Lazić et al. (2021)	Article	147	147	42	Serbia	Primary	9–18 weeks	<31 people	>5 people	Geo	3	Cognitive	Grade
Rijken and Fraser (2023)	Article	284	284	73	Australia	Middle	>18 weeks	<31 people	–	O	2	Non-cognitive	Attitude
Rijken and Fraser (2023)	Article	284	284	73	Australia	Middle	>18 weeks	<31 people	–	O	2	Non-cognitive	Attitude
Rijken and Fraser (2023)	Article	284	284	73	Australia	Middle	>18 weeks	<31 people	–	O	2	Cognitive	Grade
Saparbayeva et al. (2025)	Article	341	341	20	Kazakhstan	Tertiary	9–18 weeks	>50 people	≤5 people	Ana	2	Cognitive	Competency
Koparan and Güven (2014)	Article	70	70	46	Turkey	Middle	1–8 weeks	31–50 people	≤5 people	PaS	3	Cognitive	Competency
Koparan and Güven (2014)	Article	70	70	46	Turkey	Middle	1–8 weeks	31–50 people	≤5 people	PaS	3	Non-cognitive	Attitude
Tabuk and Özdemir (2009)	Article	96	96	46	Turkey	Primary	9–18 weeks	<31 people	–	PaS	0	Cognitive	Grade
Tabuk and Özdemir (2009)	Article	96	96	46	Turkey	Primary	9–18 weeks	<31 people	–	PaS	0	Cognitive	Grade
Alenezi (2023)	Article	50	50	48	Saudi Arabia	Middle	1–8 weeks	<31 people	–	Alg	3	Non-cognitive	Attitude
Alenezi (2023)	Article	50	50	48	Saudi Arabia	Middle	1–8 weeks	<31 people	–	Alg	3	Cognitive	Grade
Alenezi (2023)	Article	50	50	48	Saudi Arabia	Middle	1–8 weeks	<31 people	–	Alg	3	Cognitive	Grade
Alenezi (2023)	Article	50	50	48	Saudi Arabia	Middle	1–8 weeks	<31 people	–	Alg	3	Cognitive	Competency
Holmes and Hwang (2016)	Article	60	60	60	United States	Middle	>18 weeks	31–50 people	–	O	1	Cognitive	Grade
Del Valle-Ramón et al. (2020)	Article	40	40	67	Spain	Primary	1–8 weeks	<31 people	≤5 people	Geo	2	Cognitive	Grade
Lin et al. (2025)	Article	72	72	43	China	Primary	1–8 weeks	31–50 people	≤5 people	Geo	2	Cognitive	Grade
Lin et al. (2025)	Article	71	71	43	China	Primary	1–8 weeks	31–50 people	≤5 people	Geo	2	Cognitive	Grade
Quigley (2010)	Doctoral dissertation	44	44	60	United States	Primary	1–8 weeks	<31 people	–	Alg	0	Cognitive	Grade
Shchetynska (2020)	Doctoral dissertation	66	66	60	United States	Middle	9–18 weeks	31–50 people	–	Geo	1	Cognitive	Grade
Carter (2016)	Doctoral dissertation	122	122	60	United States	Middle	1–8 weeks	31–50 people	≤ 5 people	Ana	0	Cognitive	Grade
Carter (2016)	Doctoral dissertation	122	122	60	United States	Middle	1–8 weeks	31–50 people	≤ 5 people	Ana	0	Non-cognitive	Attitude
Carter (2016)	Doctoral dissertation	122	122	60	United States	Middle	1–8 weeks	31–50 people	≤5 people	Ana	0	Non-cognitive	Attitude
Carter (2016)	Doctoral dissertation	122	122	60	United States	Middle	1–8 weeks	31–50 people	≤5 people	Ana	0	Non-cognitive	Attitude
Carter (2016)	Doctoral dissertation	122	122	60	United States	Middle	1–8 weeks	31–50 people	≤5 people	Ana	0	Non-cognitive	Attitude
Garner (2020)	Doctoral dissertation	755	755	60	United States	Middle	>18 weeks	>50 people	–	O	0	Cognitive	Grade
Sahli (2017)	Doctoral dissertation	3,986	3,986	60	United States	Primary	>18 weeks	–	–	O	4	Cognitive	Grade
Sahli (2017)	Doctoral dissertation	3,390	3,390	60	United States	Primary	>18 weeks	–	–	O	4	Cognitive	Grade
Sahli (2017)	Doctoral dissertation	4,088	4,088	60	United States	Primary	>18 weeks	–	–	O	4	Cognitive	Grade
Sahli (2017)	Doctoral dissertation	3,154	3,154	60	United States	Middle	>18 weeks	–	–	O	4	Cognitive	Grade
Sahli (2017)	Doctoral dissertation	2,826	2,826	60	United States	Middle	>18 weeks	–	–	O	4	Cognitive	Grade
Wang and Kustsevalova (2023)	Conference paper	44	44	60	China	Tertiary	9–18 weeks	<31 people	–	Ana	3	Cognitive	Grade
Cervantes et al. (2015)	Article	227	227	60	United States	Middle	>18 weeks	–	–	Alg	0	Cognitive	Grade
Cervantes et al. (2015)	Article	227	227	60	United States	Middle	>18 weeks	–	–	Alg	1	Cognitive	Grade
Cervantes et al. (2015)	Article	227	227	60	United States	Middle	>18 weeks	–	–	Geo	1	Cognitive	Grade
Cervantes et al. (2015)	Article	227	227	60	United States	Middle	>18 weeks	–	–	Geo	1	Cognitive	Grade
Cervantes et al. (2015)	Article	227	227	60	United States	Middle	>18 weeks	–	–	PaS	1	Cognitive	Grade
Cervantes et al. (2015)	Article	234	234	60	United States	Middle	>18 weeks	–	–	Alg	1	Cognitive	Grade
Cervantes et al. (2015)	Article	234	234	60	United States	Middle	>18 weeks	–	–	Alg	1	Cognitive	Grade
Cervantes et al. (2015)	Article	234	234	60	United States	Middle	>18 weeks	–	–	Geo	1	Cognitive	Grade
Cervantes et al. (2015)	Article	234	234	60	United States	Middle	>18 weeks	–	–	Geo	1	Cognitive	Grade
Cervantes et al. (2015)	Article	234	234	60	United States	Middle	>18 weeks	–	–	PaS	1	Cognitive	Grade
Rehman et al. (2023)	Article	70	70	5	Pakistan	Primary	1–8 weeks	31–50 people	>5 people	O	1	Cognitive	Competency
Al Labadi and Ly (2025)	Article	209	209	72	China	Tertiary	9–18 weeks	>50 people	>5 people	PaS	4	Cognitive	Grade
Al Labadi and Ly (2025)	Article	207	207	72	China	Tertiary	9–18 weeks	>50 people	>5 people	PaS	4	Cognitive	Grade
Al Labadi and Ly (2025)	Article	199	199	72	China	Tertiary	9–18 weeks	>50 people	>5 people	PaS	4	Cognitive	Grade
Al Labadi and Ly (2025)	Article	194	194	72	China	Tertiary	9–18 weeks	>50 people	>5 people	PaS	4	Cognitive	Grade
Peker Ünal (2018)	Article	92	92	46	Turkey	Middle	1–8 weeks	<31 people	–	Alg	2	Cognitive	Grade
Özdemir et al. (2015)	Article	70	70	46	Turkey	Middle	1–8 weeks	31–50 people	≤5 people	Alg	2	Cognitive	Grade
Özdemir et al. (2015)	Article	70	70	46	Turkey	Middle	1–8 weeks	31–50 people	≤5 people	Alg	2	Non-cognitive	Attitude
Ndia et al. (2020)	Article	62	62	5	IndonesiaIndonesia	Middle	9–18 weeks	31–50 people	–	O	2	Cognitive	Grade
Condori Condori (2024)	Master’s thesis	62	62	20	Peru	Middle	1–8 weeks	31–50 people	≤5 people	O	0	Cognitive	Grade
Holmes (2022)	Doctoral dissertation	136	136	60	United States	Primary	>18 weeks	>50 people	–	O	0	Cognitive	Grade
Li (2022)	Master’s thesis	108	108	43	China	Middle	1–8 weeks	>50 people	>5 people	PaS	1	Cognitive	Grade
Li (2022)	Master’s thesis	108	108	43	China	Middle	1–8 weeks	>50 people	>5 people	PaS	1	Non-cognitive	Attitude
Wang (2022)	Master’s thesis	117	117	43	China	Middle	1–8 weeks	>50 people	>5 people	Alg	2	Cognitive	Grade
Wang (2022)	Master’s thesis	112	112	43	China	Middle	1–8 weeks	>50 people	>5 people	Alg	2	Non-cognitive	Attitude
Wang (2022)	Master’s thesis	112	112	43	China	Middle	1–8 weeks	>50 people	>5 people	Alg	2	Non-cognitive	Attitude
Wang (2022)	Master’s thesis	112	112	43	China	Middle	1–8 weeks	>50 people	>5 people	Alg	2	Non-cognitive	Attitude
Ma (2024)	Master’s thesis	97	97	43	China	Middle	1–8 weeks	31–50 people	≤5 people	Geo	3	Cognitive	Grade
Ma (2024)	Master’s thesis	97	97	43	China	Middle	1–8 weeks	31–50 people	≤5 people	Geo	3	Non-cognitive	Attitude
Ma (2024)	Master’s thesis	97	97	43	China	Middle	1–8 weeks	31–50 people	≤5 people	Geo	3	Non-cognitive	Attitude
Ma (2024)	Master’s thesis	97	97	43	China	Middle	1–8 weeks	31–50 people	≤5 people	Geo	3	Non-cognitive	Attitude
Ma (2024)	Master’s thesis	97	97	43	China	Middle	1–8 weeks	31–50 people	≤5 people	Geo	3	Non-cognitive	Attitude
Feng (2024)	Master’s thesis	112	112	43	China	Middle	1–8 weeks	>50 people	>5 people	Geo	0	Cognitive	Grade
Feng (2024)	Master’s thesis	103	103	43	China	Middle	1–8 weeks	>50 people	>5 people	Geo	0	Non-cognitive	Attitude
Zhang (2016)	Master’s thesis	85	85	43	China	Primary	9–18 weeks	31–50 people	–	O	3	Cognitive	Grade
Zhang (2016)	Master’s thesis	85	85	43	China	Primary	9–18 weeks	31–50 people	–	O	3	Non-cognitive	Attitude
Zhang (2016)	Master’s thesis	85	85	43	China	Primary	9–18 weeks	31–50 people	–	O	3	Cognitive	Competency
Zhang (2016)	Master’s thesis	85	85	43	China	Primary	9–18 weeks	31–50 people	–	O	3	Cognitive	Competency
Xu (2025)	Master’s thesis	114	114	43	China	Middle	1–8 weeks	>50 people	–	O	3	Cognitive	Grade
Chen (2024)	Master’s thesis	91	91	43	China	Middle	1–8 weeks	31–50 people	–	O	3	Cognitive	Grade
Qiu (2023)	Master’s thesis	83	83	43	China	Middle	9–18 weeks	31–50 people	>5 people	O	2	Cognitive	Grade
Zhao (2023)	Master’s thesis	96	96	43	China	Middle	1–8 weeks	31–50 people	>5 people	Geo	3	Cognitive	Grade
Zhang X. H. (2024)	Master’s thesis	101	101	43	China	Middle	1–8 weeks	31–50 people	>5 people	O	3	Cognitive	Grade
Zhang X. H. (2024)	Master’s thesis	101	101	43	China	Middle	1–8 weeks	31–50 people	>5 people	O	3	Non-cognitive	Attitude
Zhang X. H. (2024)	Master’s thesis	101	101	43	China	Middle	1–8 weeks	31–50 people	>5 people	O	3	Non-cognitive	Attitude
Zhang X. H. (2024)	Master’s thesis	101	101	43	China	Middle	1–8 weeks	31–50 people	>5 people	O	3	Non-cognitive	Attitude
Lyu (2023)	Master’s thesis	80	80	43	China	Primary	1–8 weeks	31–50 people	≤5 people	O	2	Cognitive	Competency
Lyu (2023)	Master’s thesis	80	80	43	China	Primary	1–8 weeks	31–50 people	≤5 people	O	2	Cognitive	Competency
Lyu (2023)	Master’s thesis	80	80	43	China	Primary	1–8 weeks	31–50 people	≤5 people	O	2	Cognitive	Competency
Lyu (2023)	Master’s thesis	80	80	43	China	Primary	1–8 weeks	31–50 people	≤5 people	O	2	Cognitive	Competency
Zhang W. J. (2024)	Master’s thesis	106	106	43	China	Middle	1–8 weeks	>50 people	>5 people	Geo	4	Cognitive	Grade
Zhang W. J. (2024)	Master’s thesis	106	106	43	China	Middle	1–8 weeks	>50 people	>5 people	Geo	4	Non-cognitive	Attitude
Zhang W. J. (2024)	Master’s thesis	106	106	43	China	Middle	1–8 weeks	>50 people	>5 people	Geo	4	Non-cognitive	Attitude
Zhang W. J. (2024)	Master’s thesis	106	106	43	China	Middle	1–8 weeks	>50 people	>5 people	Geo	4	Non-cognitive	Attitude
Zhang W. J. (2024)	Master’s thesis	106	106	43	China	Middle	1–8 weeks	>50 people	>5 people	Geo	4	Non-cognitive	Attitude

PT, Paper type; CB, Cultural Background; EL, Educational level; ID, Intervention Duration; GS, Group Size; CS, Class Size; TMK, Types of Mathematical Knowledge; NTT, Number of Technology Types; TLP, Learning Performance; Alg, Algebraic Knowledge; Ana, Analytical Knowledge; Geo, Geometric Knowledge; PaS, Knowledge of probability and statistics; O, Others; TLP, Type of learning performance.

### 3.4 Assessment of study quality

Two reviewers independently assessed the quality of the 35 studies using the quality index checklist developed by Montuori et al. (2024) and Nie et al. (2025) (see Table 3). The checklist comprises seven items, each scored as one point, for a total possible score of 0 to 7. Overall, the study quality ranged from moderate to high. Inter-rater reliability was excellent (Cohen’s Kappa = 0.977, *p* < 0.001).

**Table 3:** Quality of the original studies included in the meta-analysis.

ID	Author(s)/year	True RCT or matched design	Active control group	Pre–post test measures	Reliability of the assessment tool	Pretest equivalence	Method clarity	Is the intervention sufficiently explained?	Final quality score (max. 7)	Percentage of indicators present
1	Lazić et al. (2021)	0	1	1	1	1	1	1	6	85.71%
2	Rijken and Fraser (2023)	0	1	1	1	0	1	0	4	57.14%
3	Saparbayeva et al. (2025)	0	1	1	1	1	1	1	6	85.71%
4	Koparan and Güven (2014)	0	1	1	1	1	1	1	6	85.71%
5	Tabuk and Özdemir (2009)	0	1	1	1	1	1	1	6	85.71%
6	Alenezi (2023)	0	1	1	0	0	1	1	4	57.14%
7	Holmes and Hwang (2016)	0	1	1	1	1	1	1	6	85.71%
8	Del Valle-Ramón et al. (2020)	0	1	1	0	1	1	1	6	85.71%
9	Lin et al. (2025)	0	1	1	1	1	1	1	6	85.71%
10	Quigley (2010)	0	1	1	1	1	1	1	6	85.71%
11	Shchetynska (2020)	0	1	1	1	0	1	1	5	71.43%
12	Carter (2016)	0	1	1	1	0	1	1	5	71.43%
13	Garner (2020)	0	1	0	1	0	1	1	4	57.14%
14	Sahli (2017)	0	1	1	1	0	1	1	5	71.43%
15	Wang and Kustsevalova (2023)	0	1	1	1	0	1	1	5	71.43%
16	Cervantes et al. (2015)	0	1	1	1	1	1	1	6	85.71%
17	Rehman et al. (2023)	0	1	1	1	1	1	1	6	85.71%
18	Al Labadi and Ly (2025)	0	1	0	1	0	1	1	4	57.14%
19	Peker Ünal (2018)	0	1	1	0	0	1	1	4	57.14%
20	Özdemir et al. (2015)	0	1	1	1	1	1	1	6	85.71%
21	Ndia et al. (2020)	0	1	1	1	1	1	1	6	85.71%
22	Condori Condori (2024)	0	1	1	1	1	1	1	6	85.71%
23	Holmes (2022)	0	1	0	1	0	1	1	4	57.14%
24	Li (2022)	0	1	1	1	1	1	0	5	71.43%
25	Wang (2022)	0	1	0	1	1	1	1	5	71.43%
26	Ma (2024)	0	1	1	1	1	1	0	5	71.43%
27	Feng (2024)	0	1	1	1	1	1	0	5	71.43%
28	Zhang (2016)	0	1	1	1	1	1	1	6	85.71%
29	Xu (2025)	0	1	0	1	0	1	1	4	57.14%
30	Chen (2024)	0	1	1	1	1	1	1	6	85.71%
31	Qiu (2023)	0	1	1	1	0	1	1	5	71.43%
32	Zhao (2023)	0	1	1	1	1	1	1	6	85.71%
33	Zhang X. H. (2024)	0	1	1	1	1	1	1	6	85.71%
34	Lyu (2023)	0	1	1	1	1	1	1	6	85.71%
35	Zhang W. J. (2024)	0	1	1	1	1	1	1	6	85.71%

1 point for compliance, 0 points for non-compliance or inapplicability.

This study adopted the Cochrane ROB1 tool to assess the quality of all included original studies. As a classic quality assessment framework for meta-analyses of quasi-experimental studies, this tool covers seven core bias domains: confounding bias, bias in classification of interventions, selection bias, bias due to deviations from intended interventions, bias due to missing data, bias in outcome measurement, and bias due to selective reporting. In accordance with the assessment criteria for each domain, the risk of bias of each study was classified into three levels: Low, Medium, and High. The overall risk of bias of each study was then determined based on the ratings across all domains. Using the robvis package in R, we generated summary plots and traffic light plots for risk of bias. Weighted quantification was performed to characterize the distribution of bias across included studies, so as to objectively evaluate the overall quality of the literature for this meta-analysis.

### 3.5 Statistical analysis

Statistical analyses were conducted in R version 4.5.2 using the metafor package. The procedures included: (1) Effect size calculation: Cohen’s *d* was employed as the effect size index. All effect sizes from each study were incorporated to construct a three-level model. Cohen’s *d* standardizes mean differences between experimental and control groups, eliminates variation due to different instruments and units, performs robustly with large samples, and supports nested variance structures for cross-study synthesis. (2) Model selection: Random-effects models were fitted using restricted maximum likelihood (REML). These models account for inherent heterogeneity in educational interventions (e.g., stage, culture, design), appropriately weight large samples, decompose multi-level variance, and avoid false-positive bias associated with fixed-effects models, thereby supporting reliable effect estimation and moderator analysis. (3) Heterogeneity testing: Overall heterogeneity was assessed using the Q statistic. The *I*^2^ index decomposed total variance into Level 1 (sampling error), Level 2 (within-study variance), and Level 3 (between-study variance). Log-likelihood ratio tests compared model fit between the three-level model and two-level models with either Level 2 or Level 3 variance constrained to zero. (4) Moderator analysis: Subgroup analyses under mixed-effects models were conducted for categorical moderators (e.g., stage, duration), with *Q* tests assessing between-group significance. Meta-regression was applied to continuous moderators. (5) Publication bias: Funnel plot symmetry, multilevel Egger’s regression, trim-and-fill procedures, and fail-safe *N* were used to evaluate bias and the stability of results. (6) Sensitivity analysis: To evaluate the robustness of the present meta-analytic results, the leave-one-out method and the three-level Cook’s distance method were adopted in this study to exclude outliers that might substantially influence the meta-analytic outcomes. A three-level meta-analysis was then reperformed to assess the impacts of outlying effect sizes and primary studies. For the leave-one-out method, each included effect size and primary study was sequentially removed, followed by repeated three-level meta-analyses until all effect sizes and primary studies had been excluded one by one. As for the Cook’s distances method, effect sizes and primary studies with a Cook’s distance greater than *4/(n − k − 1)* were eliminated, and separate three-level meta-analyses were rerun accordingly (Van den Noortgate et al., 2012).

## 4 Results

### 4.1 Assessment of risk of bias in studies

**Figure 2:**
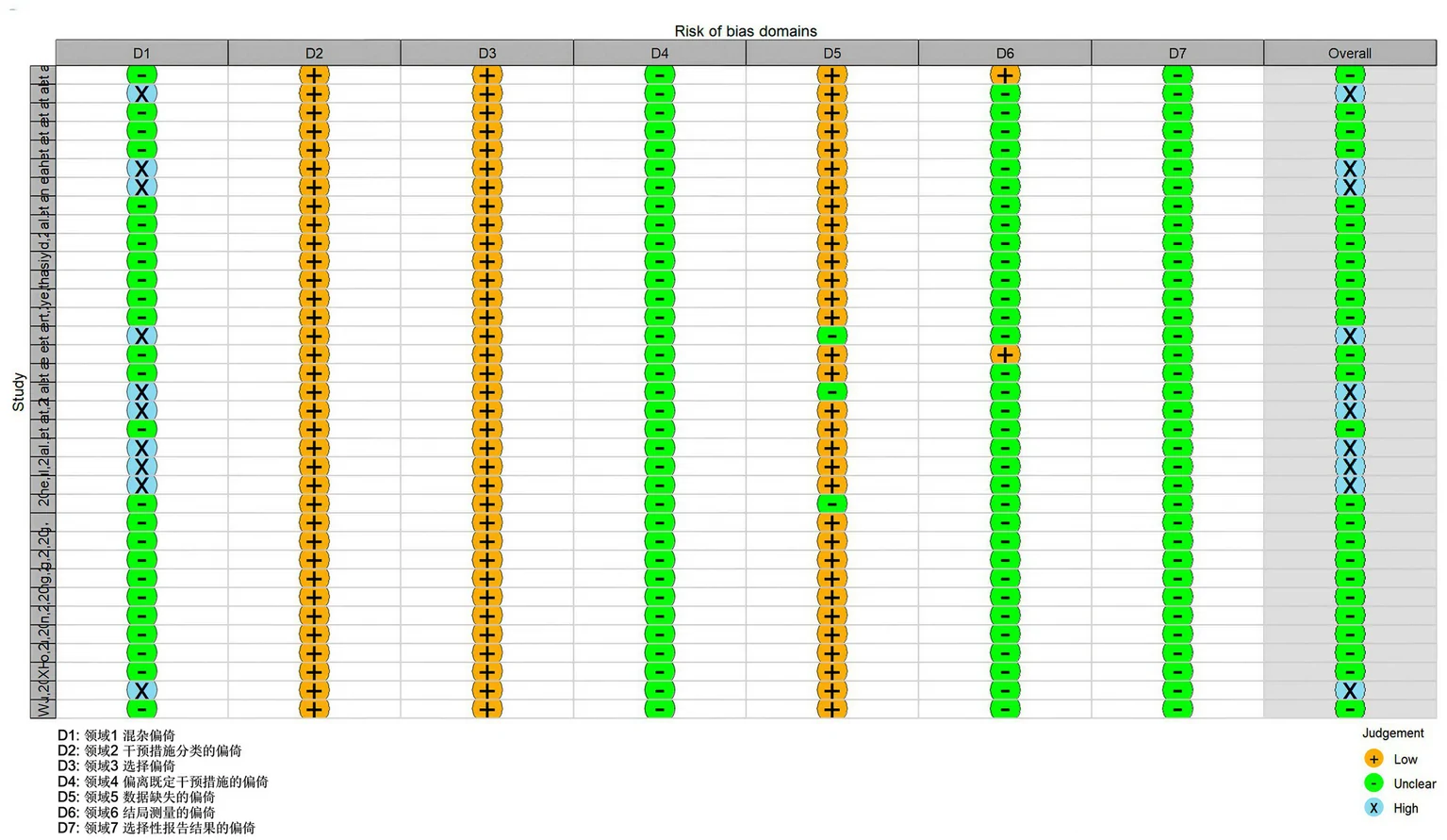
Traffic light plot.

**Figure 3:**
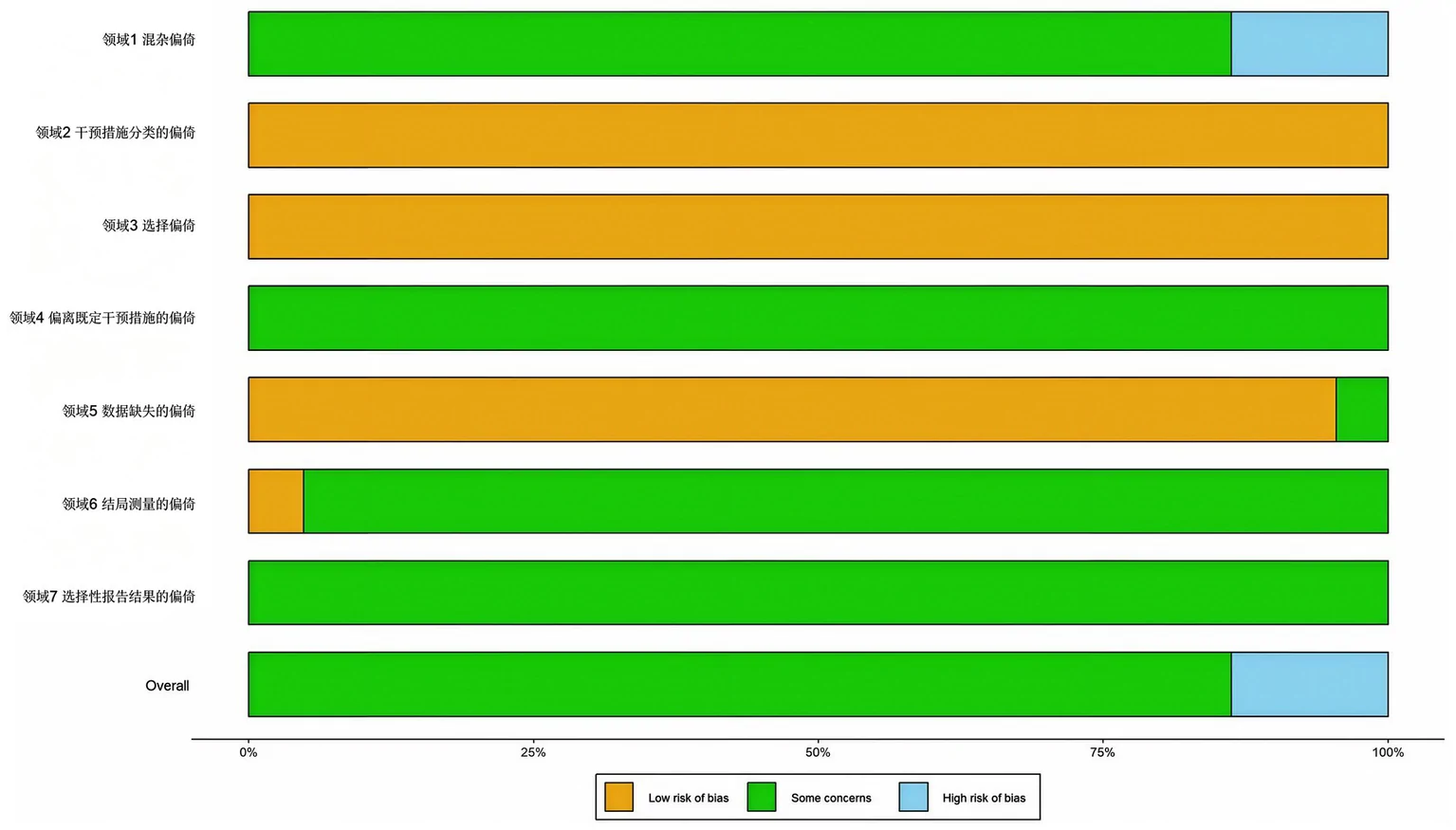
Summary plots.

A total of 35 original studies were included in this research. The ROB1 tool was used to assess the risk of bias across seven domains. Combined with the visualized results after weighted scoring, the overall risk of bias of the included literature was acceptable, and no studies presented severe methodological flaws. The distribution of bias across each domain is detailed as follows: all included studies were rated as low risk for bias in classification of interventions and selection bias, indicating rigorous literature screening and standardized definition of intervention variables, with no systematic bias caused by baseline selection or intervention grouping. Bias due to deviations from intended interventions and selective reporting of outcomes were rated as moderate risk across all studies, constituting the primary sources of bias in the included literature. A small number of studies had a high risk of bias, with several studies showing high risk in confounding bias, missing data bias, and outcome measurement bias. In total, 10 studies were identified as high-risk literature, while the remaining 25 were at moderate overall risk of bias. No low-quality studies with high risk across all domains were found. Weighted pooled results revealed that moderate bias risk was the predominant characteristic of the included literature, and the overall quality of the literature met the basic requirements for pooled meta-analysis (see Figures 2, 3).

### 4.2 Publication bias and sensitivity analyses

Two effect sizes from Del Valle-Ramón et al. (2020) and Ndia et al. (2020) exceeded the three-level Cook’s distance threshold. These effect sizes (Cohen’s *d* = 3.2825 and 2.25) were deemed statistical outliers and were removed.

**Figure 4:**
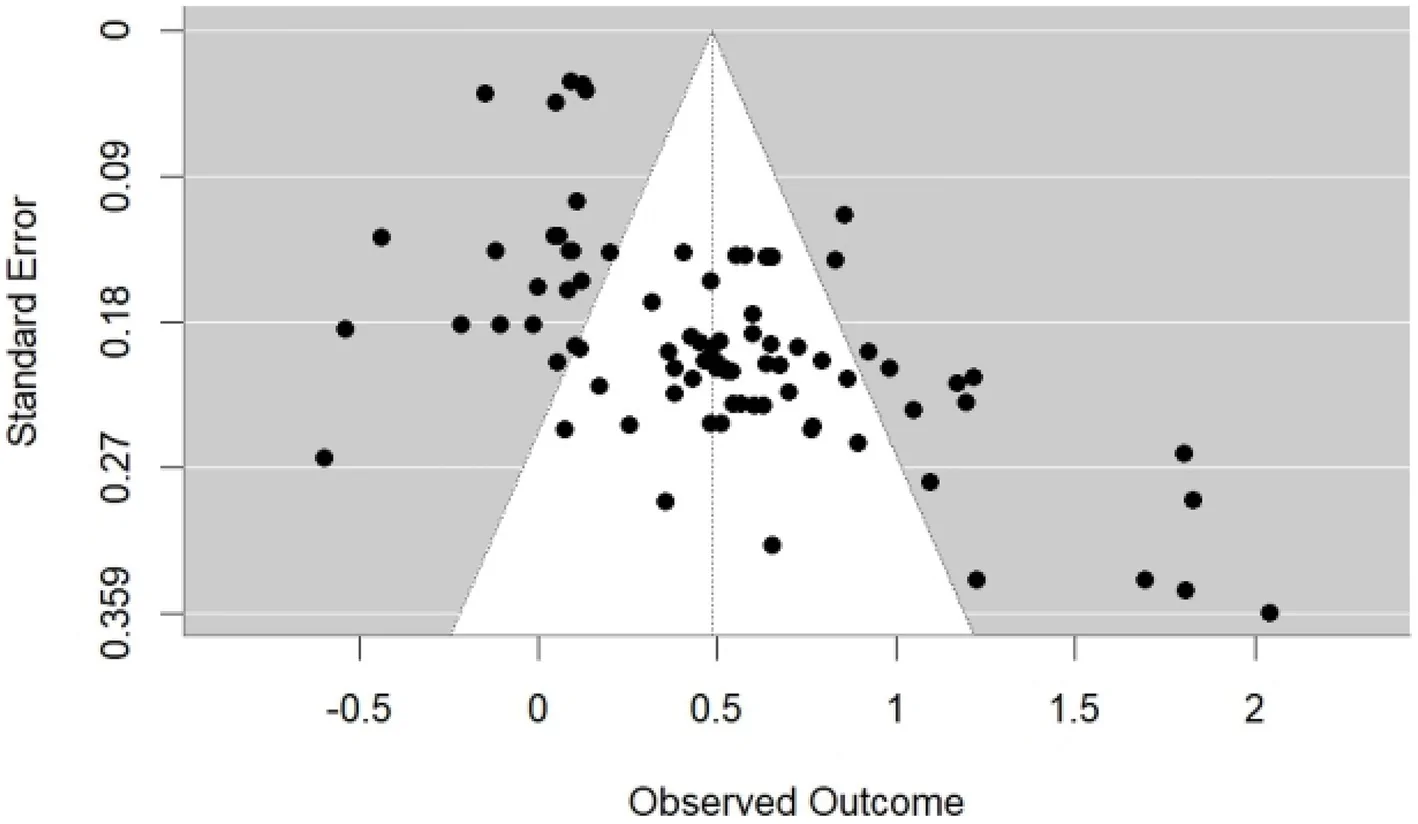
Funnel plot demonstrating publication bias in research on the effects of Project-Based Learning effectively on students’ Mathematics Learning Outcomes. The filled circles represent the distribution of the standard error of effect sizes from each study included in this meta-analysis.

After removing the two outliers, the final analysis included 33 studies and 84 effect sizes. Funnel plots (Figure 4) exhibited imperfect symmetry, with a deficit on the left side, suggesting potential publication bias. Multilevel Egger’s regression was significant (*β* = 4.56, *z* = 5.234, *p* < 0.001), indicating notable small-study effects. The trim-and-fill method estimated 23 missing studies on the left; the adjusted overall effect size was Cohen’s *d* = 0.275 (95% CI [0.157, 0.394]), suggesting a possible overestimation in the original estimate. However, this result should be interpreted cautiously due to the assumptions underlying the trim-and-fill method. The fail-safe N was 10,932, far exceeding the critical value (5 *k* + 10), supporting the robustness of the results.

Leave-one-out analyses revealed effect sizes ranging from 0.500 to 0.550 after sequential deletion, confirming minimal influence of individual studies or effect sizes and stable overall conclusions. After removing all 17 theses/dissertations and retaining only the 18 peer-reviewed journal articles, the pooled effect size was *d* = 0.487 (95% CI [0.31, 0.66]), close to the original estimate (0.529), indicating that the inclusion of gray literature did not substantially bias the results.

### 4.3 Main effects and heterogeneity

**Figure 5:**
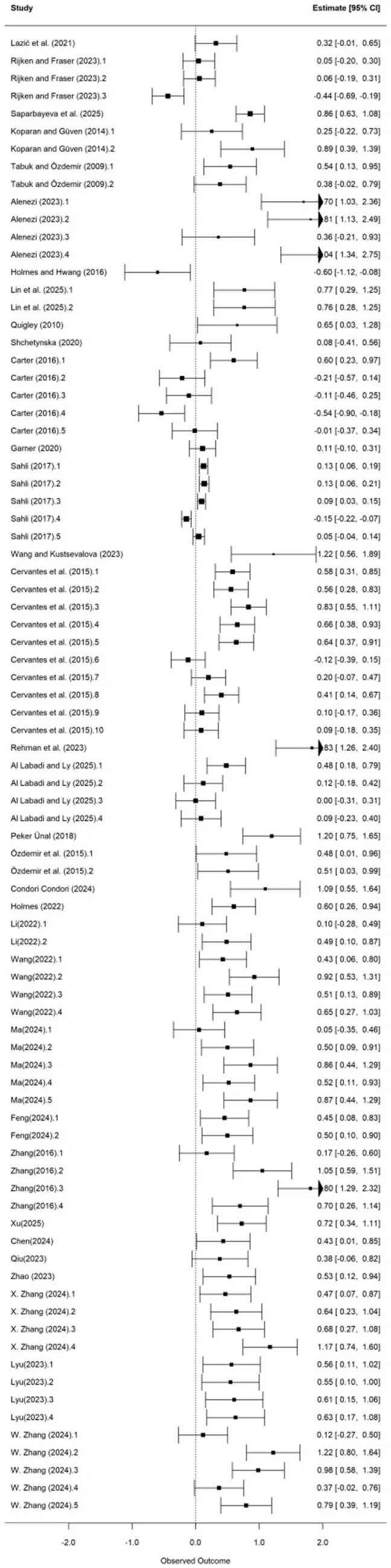
A forest plot based on a random-effects model showing the effects of Project-Based Learning Effectively on Students’ Mathematics Learning Outcomes. Cohen’s *d* is used to illustrate the effect magnitude and confidence interval in the figure. *d* = 0.529 is the composite effect size.

Three-level random-effects modeling revealed a significant overall positive effect of PBL on mathematics learning outcomes (Cohen’s *d* = 0.529, 95% CI [0.38, 0.68], *t* = 7.14, *p* < 0.001), representing a medium to large effect according to Cohen’s (1992) criteria. Model fit statistics were as follows: AIC = 104.584, BIC = 111.841. Forest plots are presented in Figure 5.

Heterogeneity testing confirmed significant variation across effect sizes (*Q*(83) = 659.185, *p* < 0.001). Log-likelihood ratio tests demonstrated a significantly better fit for the three-level model compared to two-level models with either Level 2 (*χ*^2^ = 78.609, *p* < 0.001) or Level 3 (*χ*^2^ = 17.895, *p* < 0.001) variance constrained to zero, thereby validating the three-level specification: effect sizes varied significantly both within and between studies. Variance decomposition revealed that 37.19% of the total variance (*I*^2^_level2 = 37.19%) was attributable to within-study variation (*σ*^2^ = 0.076, *p* < 0.001), while 55.53% (*I*^2^_level3 = 55.53%) was due to between-study variation (*σ*^2^ = 0.113, *p* < 0.001). According to Higgins et al. (2003), within-study heterogeneity was mild, whereas between-study heterogeneity was substantial, indicating a strong influence of study-level characteristics on effect sizes.

Regarding Research Question 1, subgroup analyses by outcome type revealed that PBL had a significantly stronger effect on non-cognitive abilities (Cohen’s *d* = 0.605, 95% CI [0.31, 0.90], *p* < 0.001) than on cognitive abilities (Cohen’s *d* = 0.581, 95% CI [0.38, 0.78], *p* < 0.001) (see Table 4).

**Table 4:** Comprehensive effect estimates of project-based learning effectively on students’ mathematics learning outcomes.

Outcome measure	*k*	#es	*N*	*t*	Cohen’s *d*	95% CI	*Q_E_*
Mathematics learning outcomes	33	84	27, 999	7.14^***^	0.529	[0.38, 0.68]	659.19
Cognitive abilities	33	60	25, 108	5.80^***^	0.581	[0.38, 0.78]	537.40
Non-cognitive abilities	11	26	2, 993	4.22^***^	0.605	[0.31, 0.90]	150.57

*k*, number of studies; # es, number of effect sizes; *t*, *t* test value for the difference between mean effect size and 0; Cohen’s *d*, effect size; CI, confidence interval; ^*^p < 0.05; ^**^p < 0.01; ^***^p < 0.001.

### 4.4 Moderator effects

To explore sources of heterogeneity, moderator analyses were conducted (see Table 5). Both cultural background and intervention duration demonstrated significant moderating effects.

**Table 5:** Moderating effect test of project-based learning effectively on students’ mathematics learning outcomes.

Moderator variable	*β*_0_	95% CIs	*t*_0_	*β*_1_	95% CIs	*t*_1_	*F* (df_1_, df_2_)
Cultural background				−0.022	[−0.03, −0.01]	−5.44	*F* (1, 82) = 29.56***
Number of technology types				0.037	[−0.07, 0.15]	0.66	*F* (1, 82) = 0.44
Publication type							*F* (1, 82) = 0.179
Journal article	0.545	[0.349,0.741]	5.538				
Dissertation and other materials	0.488	[0.308,0.699]	5.383	−0.051	[−0.290,0.188]	−0.423	
Technology use							*F* (1, 82) = 0.15
No Technology	0.478	[0.18, 0.78]	3.18**				
With Technology	0.541	[0.38, 0.70]	6.67***	0.063	[−0.26, 0.39]	0.39	
Education level							*F* (2, 81) = 0.95
Primary	0.662	[0.40, 0.93]	5.00^***^				
Secondary	0.467	[0.29, 0.64]	5.29^***^	−0.195	[−0.49, 0.10]	−1.33	
University	0.620	[0.11, 1.13]	2.40^*^	−0.042	[−0.62, 0.54]	−0.15	
Intervention duration							*F* (2, 81) = 5.33^**^
1–8 Weeks	0.666	[0.49, 0.84]	7.65^***^				
9–18 Weeks	0.528	[0.25,0.81]	3.76^***^	−0.137	[−0.47, 0.19]	−0.83	
>18 Weeks	0.110	[−0.18, 0.40]	0.76	−0.555	[−0.89,-0.22]	−3.26^**^	
Class size							*F* (2, 66) = 0.35
Small (≤30)	0.684	[0.33, 1.04]	3.87^***^				
Middle (31–50)	0.546	[0.31,0.78]	4.70^***^	−0.14	[−0.56, 0.28]	−0.66	
Large (>50)	0.496	[0.21, 0.78]	3.45^***^	−0.19	[−0.64,0.27]	−0.82^**^	
Group size							*F* (1, 45) = 0.007
≤5 Students	0.546	[0.30, 0.79]	4.54^***^				
>5 Students	0.559	[0.34,0.78]	5.13^***^	0.01	[−0.31, 0.34]	0.08	
Mathematical knowledge type							*F* (4, 79) = 0.34
Algebra	0.635	[0.34, 0.93]	4.29^***^				
Geometry	0.596	[0.34,0.78]	4.48^***^	−0.04	[−0.37, 0.30]	−0.23	
Statistics	0.439	[0.11, 0.76]	2.69^**^	−0.20	[−0.59, 0.19]	−0.99	
Analysis	0.500	[−0.01, 1.01]	1.95	−0.14	[−0.72, 0.45]	−0.46	
Others	0.483	[0.24, 0.72]	3.96^***^	−0.15	[−0.53, 0.23]	−0.80	
Outcome type							*F* (1, 82) = 3.08
Cognitive	0.484	[0.32, 0.64]	5.98^***^				
Non-cognitive	0.669	[0.45,0.89]	2.88^**^	0.19	[−0.02,0.40]	1.76	

For the moderator variables in the table, the first item is the reference category. *F* (df1, df2), the result of the omnibus test; *β*_0_, the mean effect size of effect sizes; *t*_0_, *t* test value for the difference between mean effect size and 0. *β*_1_, estimated regression coefficient; *t*_1_, *t* test value for the difference between mean effect size and reference category. ^*^p < 0.05; ^**^p < 0.01; ^***^p < 0.001.

Regarding Research Question 2:

Intervention duration significantly moderated effect sizes (*F*(2, 81) = 5.33, *p* < 0.01). Short-term interventions lasting 1–8 weeks produced the strongest effect (Cohen’s *d* = 0.666), whereas interventions exceeding 18 weeks demonstrated the weakest effect (Cohen’s *d* = 0.110).

Meta-regression revealed a significant negative predictive effect of the individualism–collectivism index (*F*(1, 82) = 29.56, *β* = −0.022, *p* < 0.01), indicating that lower scores (reflecting stronger collectivism) predicted greater PBL effects on mathematics learning.

Other moderators—educational stage, class size, group size, type of mathematical knowledge, and number of technology types—showed no significant effects (*p* > 0.05).

## 5 Discussion

### 5.1 Effects of PBL on mathematics learning: cognitive and non-cognitive outcomes

Meta-analytic results indicate that PBL enhances students’ mathematics learning (Cohen’s *d* = 0.529), consistent with prior empirical and review studies (Juandi et al., 2025). These findings further support PBL as a reform model for mathematics instruction. However, given the evidence of publication bias (trim-and-fill corrected estimate = 0.275), it cannot be regarded as decisive evidence. PBL implementation is contingent on abundant resources such as well-equipped facilities and specialized teacher training. Given its sophisticated procedural design and time-intensive operation, disparities arising from varied educational systems and resource endowments across contexts will constrain the depth of in-class inquiry and compromise implementation fidelity, ultimately restraining the complete realization of PBL’s pedagogical merits (Sánchez-García and Angosto, 2026).

No significant difference was detected between PBL’s effects on cognitive and non-cognitive outcomes, suggesting that PBL promotes these two dimensions of mathematics learning to a comparable degree. Descriptively, non-cognitive gains (Cohen’s *d* = 0.605) were slightly higher than cognitive gains (Cohen’s *d* = 0.581).

This phenomenon invites profound reflection. As Holmes and Hwang (2016) argued, learning within PBL environments demands active student engagement via hands-on projects and collaborative activities. Such learning settings are positively correlated with students’ intrinsic motivation, which in turn facilitates academic learning outcomes. From a constructivist perspective, contextualized tasks and peer interaction embedded in PBL can effectively and rapidly foster students’ non-cognitive traits, such as learning motivation and mathematical self-efficacy. In contrast, the development of advanced cognitive competencies, including logical reasoning and mathematical modeling, requires long-term knowledge accumulation and iterative reflective practice, thus exhibiting a slower improvement rate. Additionally, all included studies adopted comprehensive PBL designs without targeted training for cognitive or non-cognitive development; this may constitute one of the contributing factors underlying this phenomenon.

Subgroup descriptive analyses indicated a graded pattern of effect sizes across specific learning indicators, with mathematical literacy yielding the largest effect, followed by learning emotion, attitude, and motivation, and academic performance showing the smallest magnitude. Nevertheless, this numerical trend lacks robust statistical evidence. The subgroup for mathematical literacy contained a limited number of studies with a small sample size and wide confidence intervals, and no formal between-group difference tests were conducted. Accordingly, this study refrains from overinterpreting such descriptive disparities. Future meta-analyses with more empirical studies focusing on mathematical literacy are needed to systematically compare PBL’s intervention effects across different mathematical learning indicators.

### 5.2 Moderating effects of intervention duration and cultural background

Intervention duration: According to the reciprocal determinism of social cognitive theory, human behavior is neither determined merely by external incentives and sanctions nor fully governed by internal disposition. Instead, it stems from mutual interactions among personal cognition, behavior, and environment. Learners are agentic; they adjust their actions via reflection and self-regulation, and their behaviors reciprocally reshape the learning environment. Applied to project-based learning, short intensive interventions (1–8 weeks) foster a virtuous tripartite cycle. Within a condensed timeline, students gain periodic mastery experiences and sustain active inquiry through self-regulation; proactive behaviors improve environmental conditions, which further reinforce learning engagement and produce robust learning outcomes (Schunk, 2012; Schunk and DiBenedetto, 2020).

By contrast, when project-based learning lasts more than 18 weeks, this dynamic equilibrium breaks down. Extended projects continuously consume learners’ finite cognitive resources and increase demands on self-regulation. Over time, learners struggle to maintain in-depth inquiry and resort to superficial, passive learning strategies. These disengaged behaviors hinder environmental improvement, and the suboptimal learning context further undermines sustained active exploration. The resultant vicious cycle triggers chronic cognitive overload and gradually erodes the instructional benefits of project-based learning (Schunk and DiBenedetto, 2020).

An important interpretive caveat merits explicit clarification: the foregoing outcomes only characterize the empirical sample included in this meta-analysis and solely indicate that robust positive effects of long-cycle project-based interventions could not be reliably identified in the current dataset. These findings do not rule out the possibility that carefully refined long-form PBL curricula with differentiated supports and streamlined task structures may still yield tangible beneficial outcomes for students’ mathematical development. Future research can redress the limitations associated with short-term outcome measurements via large-scale empirical investigations and protracted longitudinal tracking. Lin et al. (2025) also noted that the duration of experimental interventions may relate to variations in learning outcomes. Subsequent meta-analytic syntheses could include a greater number of long-term intervention studies to draw more dependable conclusions about the enduring instructional effects of PBL.

Cultural background: Higher collectivism orientation predicted larger intervention effect sizes in PBL implementation. This cross-cultural moderating pattern suggests that cultural value orientations may shape the magnitude of pedagogical benefits derived from inquiry-based learning.

Collaborative teamwork lies at the core of PBL, as students work collectively to tackle authentic real-world mathematical problems (Rehman et al., 2023). Learners raised in collectivist cultural contexts generally embrace both mastery goals and group-oriented achievement goals simultaneously. When participating in collaborative mathematical PBL activities, these students strive to strengthen their logical thinking skills, attach great importance to group outcomes, and are willing to split complex cognitive tasks and exchange ideas with peers reciprocally. Such motivational differences further shape the overall instructional effectiveness of PBL. Achievement Goal Theory (AGT) posits that within achievement-related contexts, including educational environments, individuals’ motivation and achievement-relevant behaviors are influenced by their goal orientations, which define how learners perceive success and assess their own competence (Jiang and Zhang, 2021).

Prior scholarship has examined how cultural contexts shape students’ motivational orientations (Struck Jannini et al., 2024). As Liu (2021) demonstrated, culture may influence students’ mindsets, their adoption of achievement goals, as well as the correlational relationships linking these goals to motivation and academic outcomes. The instructional potency of project-based learning hinges fundamentally on sustained, deep collaborative inquiry. Group-aligned achievement motives facilitate full engagement in co-constructive knowledge building, thereby amplifying the incremental benefits of inquiry pedagogy. Conversely, exclusive focus on individual performance may precipitate social loafing and perfunctory task engagement, constraining the cognitive resources allocated to rigorous mathematical reasoning and attenuating the relative advantage of PBL over didactic lecture instruction.

While the individualism–collectivism index was a significant predictor, readers should interpret this finding with caution. The index is measured at the national level and may not capture classroom- or school-level cultural variations. Moreover, differences in national curricula, teacher training, implementation fidelity, and instructional resources across countries could confound the observed cultural effect. Therefore, the significant moderation by cultural background should be seen as hypothesis-generating rather than definitive causal evidence. Future research should measure cultural orientation at the individual or classroom level and control for implementation quality.

### 5.3 Non-significant moderators

The non-significant effects of educational stage, class size, group size, knowledge type, and technology count do not diminish their potential importance; several explanations may account for this.

Educational stage: The non-significant findings align with some prior research (Chen and Yang, 2019), possibly due to uneven distribution across educational stages (e.g., few tertiary-level studies) and the generalizability of PBL as an instructional philosophy across age groups. Its effectiveness depends more on developmentally appropriate project design than on the educational stage itself.

Class/group size: The lack of significant findings aligns with prior results (Chen and Yang, 2019), likely due to extensive missing data from incomplete reporting in primary studies, which reduces statistical power. Zhang and Ma (2023) identified benefits for small classes and groups of 4–5 individuals, suggesting that true effects may depend on data quality, sample characteristics, and context. These findings warrant further investigation with more comprehensive reporting.

The finding of non-significance contradicts Juandi et al. (2025), likely due to coding challenges. Many projects integrate multiple content areas and resist strict single-category classification. PBL prioritizes interdisciplinary problem-solving over isolated skill instruction. Future research should employ more detailed and operational content classifications to enhance precision.

Publication type: We grouped all included studies into journal articles, dissertations, and other publications, and examined publication type as a categorical moderator. No significant moderating effect was observed. Since publication type only reflects the publication format rather than research quality itself, the core relationship between variables proved robust across different publication channels. Despite the general consensus that peer-reviewed journal articles feature superior research quality, the resultant quality disparities did not exert a notable impact on our main results. Full details can be found in Table 5.

The number of technology types used is not a significant factor, which contrasts with the findings of Zhang and Hu (2019), although Chen and Yang (2019) supported technology-supported PBL. In mathematics education, the quantity of technology does not equate to the quality of integration. Simply adding more types of tools or novel technologies does not guarantee better outcomes. What is critical is the deep, functional integration of technology to support inquiry, collaboration, expression, and creation as cognitive tools. Teachers should prioritize the appropriateness, depth, and functionality of technology over its quantity or novelty, using it to enhance mathematical understanding, problem-solving, and higher-order thinking.

### 5.4 Practical implications

Results of the present meta-analysis indicate that mathematics teachers may implement short-term, intensive project-based learning (PBL) interventions spanning 1 to 8 weeks to modestly improve program outcomes. This preliminary inference is drawn exclusively from the aggregated data of the current meta-analysis and should not be construed as binding operational guidelines. Notably, all included studies adopted quasi-experimental designs, which are susceptible to novelty effects and may overestimate the actual efficacy of the interventions. Accordingly, future research should conduct longitudinal randomized controlled trials to corroborate the long-term sustained impacts of this PBL model. Determining the duration of project implementation hinges largely on textbook content, core knowledge objectives, and the authenticity of situated tasks. Taking geometry learning as an example, the inquiry into the areas of polygons, which covers various figures including parallelograms, triangles, and trapezoids, may require a longer instructional duration. In contrast, projects such as learning position and direction by having students act as campus tour guides involve fewer knowledge points and can be completed within fewer instructional class sessions. Consequently, teachers need to maintain flexibility when scheduling project duration to align with subject content and contextual demands, so as to enhance students’ learning outcomes.

For technology use, our finding that the number of technology types was not a significant moderator suggests that quality of integration matters more than quantity; this conclusion is limited by the fact that many primary studies did not report technology use in sufficient detail. The rapid advancement of technology exerts considerable impacts on students’ learning behaviors, motivation, and development (Noor et al., 2022). Existing studies have demonstrated that artificial intelligence-enabled tools exert growing effects on improving students’ learning outcomes (Younas et al., 2025d). Artificial intelligence technologies, such as intelligent tutoring systems and adaptive learning platforms, can significantly enhance learning personalization and student engagement (Younas et al., 2025a; Younas et al., 2025b). Featuring user-friendliness, instructional benefits, and immersive learning experiences, AI-enabled tools effectively facilitate students’ active learning and deepen their learning involvement, thereby improving knowledge acquisition (Afzaal et al., 2025). Accordingly, integrating AI tools into subsequent mathematics project-based learning practices may help optimize students’ learning experiences. Nevertheless, the classroom application of AI should be implemented prudently, with full consideration of its long-term impacts on students’ cognitive development and social–emotional skill cultivation (Younas et al., 2026). Furthermore, educators need to receive enhanced professional training, and instructional resources ought to undergo careful scrutiny (Younas et al., 2025c).

The present meta-analysis identified cultural background as a significant moderator of PBL effectiveness in mathematics learning. In light of this finding, mathematics teachers may pay due attention to cross-cultural differences when designing and delivering PBL activities. For culturally responsive instructional adaptation, educators can first learn about students’ collaborative preferences, communication styles and engagement attitudes toward group work, rather than subjectively estimating team efficiency simply based on cultural identities (Zhang and Ma, 2023). Some students from distinct cultural settings might be reluctant to voice divergent ideas in group discussions or tend to rely heavily on higher-performing peers, which could slow down the progress of collaborative mathematical inquiry. During in-class implementation, teachers may co-establish group agreements to lower the incidence of free-riding and passive participation (Lazić et al., 2021). Practically, assigning well-defined individual responsibilities, setting milestone deadlines for subtasks, and embedding incentive frameworks into process-based assessment can likely motivate all group members to engage in mathematical reasoning, peer discussion and joint problem-solving. With these culturally informed adjustments, PBL is expected to better accommodate the learning profiles of students from diverse cultural backgrounds and deliver more favorable educational outcomes in mathematics teaching.

In addition, the implementation of PBL imposes relatively high professional requirements on frontline teachers. To guarantee the orderly operation and quality delivery of PBL mathematics lessons, practitioners may need to continuously deepen their subject-matter expertise, refine their curriculum design and instructional delivery techniques, develop diversified assessment instruments, and strengthen their ability to organize and regulate inquiry-based classrooms (Saparbayeva et al., 2025). In the PBL learning environment, teachers are encouraged to shift their role toward instructional facilitators. In this capacity, they can provide tailored scaffolding, directional prompts and cognitive guidance to support students in unpacking thematic tasks, sorting out logical frameworks, and independently constructing robust understanding of curriculum knowledge and project-centered content (Meng et al., 2023; Rehman et al., 2023).

### 5.5 Limitations and future directions

Several methodological and contextual limitations of this meta-analysis should be acknowledged when evaluating its analytical outcomes and instructional implications.

First, publication bias suggests the true effect may be smaller than our original estimate. Statistical detection and visual inspection of funnel plots indicated potential asymmetry in the included empirical records, which is a common methodological concern in educational intervention research. Although this study attempted to reduce selection bias by incorporating grey literature and adopting unrestricted language criteria during database retrieval, the final analytical sample was still dominated by Chinese and English published studies. Unpublished non-significant findings and studies reported in other languages may not be fully captured in the current dataset. In this case, the synthesized effect sizes might slightly deviate from the true population effect, suggesting that the overall PBL effectiveness observed in this meta-analysis should be interpreted conservatively.

Second, primary studies varied considerably in the richness of reported contextual and instructional information, which may limit the breadth of moderator analyses conducted in this three-level meta-analysis. Although group size was tentatively explored in the present study, partial missing data for this indicator may render the corresponding moderating results insufficiently comprehensive, which could potentially limit the generalizability of relevant findings. In addition, several theoretically critical moderators, including school resource availability, teacher training experience, instructional implementation quality, and task complexity, could not be quantitatively synthesized due to inconsistent reporting of these variables across individual studies, leaving a certain degree of cross-study heterogeneity unexplained. Beyond variable-related constraints, the included evidence was unevenly distributed across educational stages, with relatively limited empirical data from university mathematics contexts. Such unbalanced sample distribution may slightly reduce the statistical sensitivity of subgroup comparisons, which might hinder the identification of subtle stage-specific variations in PBL effectiveness.

Finally, several methodological limitations inherent to meta-analytic synthesis should be acknowledged. The empirical evidence synthesized in this study relies entirely on existing published studies, and the credibility of analytic outcomes is contingent upon the methodological design and reporting quality of primary research. Most included studies adopted quasi-experimental approaches, which may be subject to unmeasured confounding factors and novelty effects that could introduce uncertain fluctuations in effect size estimation. In addition, two outlier studies with highly atypical implementation contexts were excluded to optimize the stability of pooled results during data processing. Although this standard analytical practice helps enhance the reliability of the overall findings, it may narrow the contextual applicability of the current evidence. Future studies adopting rigorous randomized controlled trial designs are therefore warranted to further validate and supplement the present findings.

## 6 Conclusion

Employing a three-level meta-analytic approach, this study systematically synthesizes empirical findings to clarify the overall effectiveness and contextual boundaries of project-based learning (PBL) in mathematics education, advancing existing scholarly understanding of PBL implementation in mathematics instruction. The results revealed a small-to-moderate positive effect of PBL on students’ mathematics learning outcomes (Cohen’s *d* = 0.529; 95% CI [0.38, 0.68]). However, publication bias attenuated the overall effect, with the trim-and-fill corrected estimate declining to 0.275. Further analyses demonstrated that PBL yielded comparable improvements in students’ cognitive and non-cognitive mathematical outcomes with no significant statistical difference, indicating its capacity to foster balanced development of students’ comprehensive mathematical literacy. This study further identified the moderating effects of intervention duration and cultural context, which account for the heterogeneous effectiveness of mathematical PBL and partially explain the inconsistent findings observed in previous studies, thereby providing empirical evidence for judging the contextual applicability of PBL. Practically, the synthesized results offer referential implications for implementing short-cycle, context-adapted PBL in school mathematics teaching. Nevertheless, constrained by residual publication bias and inconsistent reporting quality across primary studies, the robustness of the current findings is limited. Accordingly, the interpretation and generalization of these results should be undertaken with caution, and this study serves only as a preliminary reference for future theoretical refinement and empirical exploration.

## Data Availability

The original contributions presented in the study are included in the article/Supplementary material, further inquiries can be directed to the corresponding author.
