# Supplementary material for: Can project-based learning improve students’ mathematics learning outcomes: a three-level meta-analysis
Source: Front Psychol. 2026 Sep 4;17:1868415. doi: 10.3389/fpsyg.2026.1868415 (PMC13586001; doi:10.3389/fpsyg.2026.1868415)
Supplement: Supplementary file 1 [file Data_Sheet_1.pdf]

Appendix S1:Search Code

Database	Search Code
Web of Science	(Project-based learning OR PBL OR PjBL) AND (mathematics OR math OR math education OR mathematics education)
ProQuest	(project based learning OR project-based learning OR pbl) AND (mathematics OR math OR math education OR mathematics education)
EBSCO	(project based learning OR project-based learning OR pbl) AND (mathematics OR math OR math education OR mathematics education)
Springer Link	(project based learning OR project-based learning OR pbl) AND (mathematics OR math OR math education OR mathematics education)
CNKI	项目式学习 OR 项目化学习 AND 数学

(Initial search: July 31, 2025; Update search: December 1, 2025 – January 10, 2026).

Appendix S2:Summary of risk of bias assessment for included studies using ROBINS-I V2

Study ID	D1	D2	D3	D4	D5	D6	D7	Overall	Weight
Lazić et al. (2021)	Medium	Low	Low	Medium	Low	Low	Medium	Medium	147.00
Rijken and Fraser (2024)	High	Low	Low	Medium	Low	Medium	Medium	High	284.00
Saparbayeva et al. (2025)	Medium	Low	Low	Medium	Low	Medium	Medium	Medium	341.00
Koparan and Güven (2014)	Medium	Low	Low	Medium	Low	Medium	Medium	Medium	70.00
Tabuk and Özdemir (2009)	Medium	Low	Low	Medium	Low	Medium	Medium	Medium	96.00
Alenezi (2023)	High	Low	Low	Medium	Low	Medium	Medium	High	50.00
Holmes and Hwang (2016)	High	Low	Low	Medium	Low	Medium	Medium	High	60.00
Del Valle-Ramón et al. (2020)	Medium	Low	Low	Medium	Low	Medium	Medium	Medium	40.00
Lin et al. (2025)	Medium	Low	Low	Medium	Low	Medium	Medium	Medium	71.50
Quigley (2010)	Medium	Low	Low	Medium	Low	Medium	Medium	Medium	44.00
Shchetynska (2020)	Medium	Low	Low	Medium	Low	Medium	Medium	Medium	66.00
Carter (2016)	Medium	Low	Low	Medium	Low	Medium	Medium	Medium	122.00
Garner (2020)	Medium	Low	Low	Medium	Low	Medium	Medium	Medium	755.00
Sahli (2017)	Medium	Low	Low	Medium	Low	Medium	Medium	Medium	3488.80

Wang and Kustsevalova (2023)	High	Low	Low	Medium	Medium	Medium	Medium	High	44.00
Cervantes et al. (2015)	Medium	Low	Low	Medium	Low	Low	Medium	Medium	230.50
Rehman et al. (2023)	Medium	Low	Low	Medium	Low	Medium	Medium	Medium	70.00
Al Labadi and Ly (2025)	High	Low	Low	Medium	Medium	Medium	Medium	High	202.25
Peker Ünal (2018)	High	Low	Low	Medium	Low	Medium	Medium	High	92.00
Özdemir et al. (2015)	Medium	Low	Low	Medium	Low	Medium	Medium	Medium	70.00
Ndia et al. (2020)	High	Low	Low	Medium	Low	Medium	Medium	High	62.00
Condori (2024)	High	Low	Low	Medium	Low	Medium	Medium	High	62.00
Holmes (2022)	High	Low	Low	Medium	Low	Medium	Medium	High	136.00
Li (2022)	Medium	Low	Low	Medium	Medium	Medium	Medium	Medium	108.00
Wang (2022)	Medium	Low	Low	Medium	Low	Medium	Medium	Medium	113.25
Ma (2024)	Medium	Low	Low	Medium	Low	Medium	Medium	Medium	97.00
Feng (2024)	Medium	Low	Low	Medium	Low	Medium	Medium	Medium	107.50
Zhang (2016)	Medium	Low	Low	Medium	Low	Medium	Medium	Medium	85.00
Xu (2025)	Medium	Low	Low	Medium	Low	Medium	Medium	Medium	114.00

Chen (2024)	Medium	Low	Low	Medium	Low	Medium	Medium	Medium	91.00
Qiu (2023)	Medium	Low	Low	Medium	Low	Medium	Medium	Medium	83.00
Zhao (2023)	Medium	Low	Low	Medium	Low	Medium	Medium	Medium	96.00
X. Zhang (2024)	Medium	Low	Low	Medium	Low	Medium	Medium	Medium	101.00
Lyu (2023)	High	Low	Low	Medium	Low	Medium	Medium	High	80.00
W. Zhang (2024)	Medium	Low	Low	Medium	Low	Medium	Medium	Medium	106.00

Note: D1=Bias due to confounding; D2=Risk of bias in classification of interventions; D3=Risk of bias in selection of participants into the study; D4=Risk of bias due to deviations from intended interventions; D5=Risk of bias due to missing data; D6=Risk of bias arising from measurement of the outcome; D7=Risk of bias in selection of the reported result.

Appendix S3:Certificate of English editing

Certificate of English Editing

E-TRANSTAR (Beijing) Information Technology Co., Ltd.

Title

Can Project-Based Learning Improve Students' Mathematics Learning Outcomes: A Three-level Meta-analysis

Authors

Chunting Wang

Date of Editing

June 11, 2026

This document certifies that the paper listed above has been edited to ensure that the language is clear and free from errors. The edited paper has reached grade A in language evaluation for SCI journals.

The edit was performed by

Waqar Ahmed Marri

native English speaker editor of

E-transtar (Beijing) Information Technology Co., Ltd.

The intent of the author's message was not altered in any way during the editing process. The quality of the edit has been guaranteed, with the assumption that our recommended changes have been accepted and have not been altered without the knowledge of our editors.

Certificate issued by

E-transtar (Beijing) Information Technology Co., Ltd.

HIGH QUALITY
